# Supplementary material for: Relationship between dietary thiamine, riboflavin, and niacin intake and hypertension subtypes: A cross-sectional study from the 1999–2023
Source: PLoS One. 2026 Apr 24;21(4):e0335834. doi: 10.1371/journal.pone.0335834 (PMC13108754; doi:10.1371/journal.pone.0335834)
Supplement: S1 Table — ISH: Isolated systolic hypertension; IDH: Isolated diastolic hypertension; SDH: Systolic-diastolic hypertension. (DOCX) [file pone.0335834.s001.docx]

**Supplementary Table 1：Associations of vitamin intake from food and supplement sources with hypertension subtypes.**

| **Vitamin & Source** | **Hypertension Subtype** | **Q1 (Ref)** | **Q2** | **Q3** | **Q4** | **P for trend** |
| --- | --- | --- | --- | --- | --- | --- |
| Thiamine (Food) | ISH | 1 | 1.05 (0.92-1.19) | 1.02 (0.89-1.16) | 1.08 (0.94-1.23) | 0.45 |
|  | IDH | 1 | 0.95 (0.70-1.28) | 0.89 (0.65-1.21) | 0.92 (0.67-1.26) | 0.58 |
|  | SDH | 1 | 1.18 (0.95-1.46) | 1.15 (0.92-1.43) | 1.22 (0.98-1.52) | 0.18 |
| Thiamine (Supplement) | ISH | 1 | 1.01 (0.89-1.15) | 0.98 (0.86-1.12) | 1.04 (0.91-1.19) | 0.72 |
|  | IDH | 1 | 1.08 (0.80-1.45) | 0.94 (0.69-1.27) | 1.11 (0.82-1.50) | 0.89 |
|  | SDH | 1 | 1.10 (0.89-1.36) | 1.05 (0.85-1.30) | 1.15 (0.93-1.42) | 0.31 |
| Riboflavin (Food) | ISH | 1 | 1.08 (0.95-1.23) | 1.22 (1.07-1.39) | 1.20 (1.05-1.37) | 0.005 |
|  | IDH | 1 | 1.12 (0.83-1.51) | 1.15 (0.85-1.55) | 1.09 (0.80-1.48) | 0.62 |
|  | SDH | 1 | 1.30 (1.05-1.61) | 1.48 (1.20-1.83) | 1.42 (1.15-1.76) | 0.001 |
| Riboflavin (Supplement) | ISH | 1 | 1.04 (0.91-1.18) | 1.07 (0.94-1.22) | 1.11 (0.97-1.27) | 0.12 |
|  | IDH | 1 | 1.05 (0.78-1.41) | 0.97 (0.72-1.31) | 1.14 (0.85-1.53) | 0.51 |
|  | SDH | 1 | 1.25 (1.01-1.55) | 1.32 (1.07-1.63) | 1.35 (1.09-1.67) | 0.008 |
| Niacin (Food) | ISH | 1 | 1.10 (0.97-1.25) | 1.06 (0.93-1.21) | 1.09 (0.95-1.25) | 0.41 |
|  | IDH | 1 | 0.88 (0.65-1.19) | 0.92 (0.68-1.24) | 0.85 (0.62-1.16) | 0.41 |
|  | SDH | 1 | 0.95 (0.77-1.18) | 0.99 (0.80-1.23) | 1.01 (0.81-1.25) | 0.88 |
| Niacin (Supplement) | ISH | 1 | 0.98 (0.86-1.12) | 1.03 (0.90-1.17) | 1.06 (0.93-1.21) | 0.35 |
|  | IDH | 1 | 1.12 (0.83-1.51) | 1.05 (0.78-1.42) | 1.18 (0.88-1.59) | 0.47 |
|  | SDH | 1 | 1.08 (0.87-1.34) | 1.10 (0.89-1.36) | 1.13 (0.91-1.40) | 0.28 |

ISH：Isolated systolic hypertension；IDH：Isolated diastolic hypertension；SDH：Systolic-diastolic hypertensio.
