# Supplementary material for: Relationship between dietary thiamine, riboflavin, and niacin intake and hypertension subtypes: A cross-sectional study from the 1999–2023
Source: PLoS One. 2026 Apr 24;21(4):e0335834. doi: 10.1371/journal.pone.0335834 (PMC13108754; doi:10.1371/journal.pone.0335834)
Supplement: Attachment — Submitted filename: Response_to_Reviewers_auresp_2.docx [file pone.0335834.s005.docx]

**Response to Reviewers**

We extend our sincere gratitude to the editors and all reviewers for their valuable comments, which have significantly enhanced the quality of this manuscript. Reviewers' comments are presented in italics, with specific issues numbered. Our responses are indicated in red typeface, and manuscript revisions are highlighted in red.

**Comments from Reviewer #1:**

***1.I have notice some gramatical errors in some part of the manuscript. So, the author better to review and correct it before publication.***

Response:Thank you very much for your careful review and for pointing out the grammatical errors in our manuscript. We sincerely appreciate your attention to detail, which has helped us improve the language quality of our work.We have thoroughly reviewed the entire manuscript and corrected all identified grammatical issues. In addition, we engaged a native English speaker with expertise in scientific writing to carefully proofread and polish the language throughout the paper. All changes have been clearly marked in the revised manuscript using the track changes feature to facilitate your re-evaluation.We believe that the revised version now meets the high language standards expected by your journal. We are grateful for your constructive suggestion, which has undoubtedly strengthened the clarity and professionalism of our manuscript.('Revised Manuscript with Track Changes'lines 30-38;68-72;112-115;124-132)
